# Invoking Uncertainty: Parents’ Accounts for Intrusions on Medical Authority in Pediatric Neurology

**DOI:** 10.1177/00221465231194052

**Published:** 2023-10-21

**Authors:** Keith Cox

**Affiliations:** 1 UCLA, Los Angeles, CA, USA

**Keywords:** conversation analysis, medical authority, pediatric neurology, physician–family communication, uncertainty

## Abstract

In pediatric medical visits, parents may assume the role of co-caregiver with clinicians. At times, parents challenge physicians’ authority to determine diagnoses and treatments for their children. The present study uses conversation analysis to examine parents’ accounts for their intrusions on medical authority in a corpus of 35 video-recorded pediatric neurology visits for overnight video-electroencephalogram monitoring. I show how parents can exploit their legitimate role as carers to challenge medical authority. Through invoking uncertainty in contexts where they have somehow challenged medical authority, parents can account for their conduct in ways that elide direct conflict with physicians and thereby minimize damage to the physician–family partnership.

Pediatric medical visits represent a unique opportunity for studying authority. In these visits, medical authority and parental authority converge on a common goal—the child’s best interests. However, clinicians and parents do not always agree on what courses of action are best. Parents may challenge medical authority but nevertheless rely on doctors for access to the medical goods and services that they need to care for their child. Doctors may disagree with parents but nevertheless rely on them to carry out treatment plans. In these points of departure, medical authority and parental authority collide. And to the extent that legitimate authority trades, as Weber (1958) suggested, on the likelihood that one can induce others to follow specific commands, parental autonomy represents an inherent limitation on medical authority in pediatrics.

I focus on this issue in a particular context: when parents invoke uncertainty during pediatric neurology visits for overnight video-electroencephalogram (vEEG) monitoring. In what follows, I offer some background on the interplay between medical authority, clinical uncertainty, and the doctor–patient relationship. Then, after describing the data and methods employed in this study, I ask two main questions: (1) When do parents invoke uncertainty in their interactions with clinicians and (2) to what ends? Finally, after detailing the functions of uncertainty invocations, I consider what might lead parents to use uncertainty to accomplish this work.

## Background

### Medical Authority

Substantial scholarly energy has been directed at the concept of medical authority over the past 70 years (Freidson 1970; Heritage 2005, 2021; Parsons 1951; Peräkylä 1998, 2002; Stivers and Timmermans 2020; Tate 2018). This authority is derived from the relative rights to claim domains of knowledge (i.e., *epistemic rights*; Heritage and Raymond 2005) and to decide future courses of action, or *deontic rights* (Stevanovic and Peräkylä 2012). Early research concluded that due to their specialized knowledge and experience, doctors are expected to know more, entitled to know more, and indeed do know more about the biomedical nature of patients’ presenting complaints than do patients (Abbott 1988; Freidson 1970; Parsons 1951). That is, physicians have the right to claim epistemic primacy over medical knowledge. Moreover, physicians can put their knowledge into practice, exercising their deontic authority to determine treatment regimens. At the height of the mid-twentieth-century period of paternalistic medicine, subordination of the patient was believed to be appropriate, and the exercise of medical authority was viewed as necessary for the practice of medicine.

In this context, Parsons (1951) articulated his functionalist formulation of the institution of medicine as a social system wherein doctors and patients have complementary roles—doctors are expected to act in patients’ best medical interests, and patients are obligated to follow medical advice. Parsons (1951) contended that this asymmetry is unavoidable in medical encounters, and thus a degree of mutual trust is vital for effective medical care. Freidson (1970) saw this asymmetry as an inherent source of doctor–patient conflict. Each, however, asserted that successful medical care ultimately depends on subordination of the patient either outright or through persuasion. In this conceptualization of the doctor–patient relationship, being a good patient meant being a passive patient, while being a good doctor meant honoring a Hippocratic commitment to beneficence toward patients, even at the cost of patient autonomy.

However, in recent years patients in the United States have become increasingly engaged in their own care, and such engagement has come to be viewed as a patient’s responsibility (Timmermans 2020). Even the ways in which participants orient to the epistemic and deontic rights underlying medical authority have changed over time: Recent studies point to increasing negotiation with physicians over diagnosis (McArthur Hernandez 2021; Stivers and Timmermans 2017) and treatment (Bergen et al. 2018; Stivers and Timmermans 2021; Timmermans 2020). However, interactionist studies examining the exercise of epistemic and deontic rights and responses to them in doctor–patient interaction indicate that medical authority remains prominent in clinical encounters (Byrne and Long 1976; Heritage 2005, 2021; Peräkylä 1998, 2002; Pilnick and Dingwall 2011), where it is collaboratively achieved by clinicians and patients (Maynard 1991).

Although modern patients play a more active role in their medical care, studies show that they remain sensitive to the boundary between matters that fall within physicians’ domains and those that fall within their own (Gill, Halkowski, and Roberts 2001; Stivers 2005). For instance, Stivers (2005) demonstrated that parents, in particular, orient to treatment recommendations as something over which they have the right to accept (or not) on their child’s behalf, exercising their deontic authority as parents to shape future courses of action for their children. Indeed, this is one way that they can display their identity as good, competent parents (Heritage and Sefi 1992; Voysey Paun 1975). Moreover, in this context, clinicians routinely orient to parent acceptance of the treatment recommendation (or mutual agreement on an alternative) as necessary before the visit can progress to closure. Thus, parents and clinicians mutually orient to treatment recommendations as at least partially within the purview of parental deontic authority.

However, physicians and patients generally treat diagnosis as something over which clinicians have primary epistemic rights (Heritage 2005, 2021; Heritage and McArthur 2019). Patients may be inclined to disagree with clinicians’ assessments of their presenting complaints, especially “no problem” diagnoses in the context of contested illnesses such as fibromyalgia (Barker 2009), and may visit multiple providers in search of a diagnosis with which they agree. However, research on video-recorded primary care medical visits in Western societies shows that patients overwhelmingly treat the provision of diagnoses as informings by acknowledging rather than accepting or rejecting them (Heath 1992; Peräkylä 1998), thereby implicitly ratifying their physicians’ epistemic authority. Although, part of patients’ apparent deference to physicians’ diagnoses can be accounted for by how they are delivered—clinicians infrequently allow for and rarely pursue acceptance of diagnoses before advancing to the treatment recommendation phase of the visit (Stivers 2005; see also Heritage 2021).

Yet, this is not to say that patients have no influence over matters that fall within physicians’ domains. For example, patients prompt physicians to offer specific diagnostic tests rather than overtly asking for them and thereby accomplish requests without explicitly making them (Gill et al. 2001; see also Gill 2005). In deploying interactional practices designed to exert influence on matters within physicians’ domains indirectly, patients both encroach on medical authority and ratify it. Thus, while prior conceptions of the doctor–patient relationship held that being a “good” patient amounted to being a passive recipient of care, being a “good” patient today may entail being engaged in one’s care but in the right ways (i.e., those that avoid direct conflict with physicians).

### Introducing Uncertainty into the Interaction

If there were no uncertainty in medicine, then there would be no need for doctors as we know them—health care would be a relatively low-skilled enterprise not *practiced* by clinicians but *applied* by technicians, like error codes in a car are read by technicians. Doctor–patient relationships are often predicated on uncertainty: It is the uncertainty arising from unexplained symptoms or failed home remedies that leads many patients to seek care in the first place. And through deference to doctors’ professional judgment, patients ratify both the cultural authority of medical science and the social authority of its practitioners (Starr 1982).

Uncertainty can serve to empower medical authority in other ways as well. Clinicians can, for example, topicalize uncertainty related to the limits of medical science as a means of advocating for or against further testing (Pilnick and Zayts 2014), or they can present relatively ambiguous test results as either more or less certain to push for or against particular diagnoses (Stivers and Timmermans 2016). Patients too may use uncertainty in ways that empower medical authority, deploying it, for instance, as a means of displaying a lack of entitlement to medical knowledge, thus treating physicians as having primary rights to it (Gill 1998). In so doing, these displays of uncertainty embody respect for physicians’ epistemic authority.

Yet, uncertainty can also undermine medical authority. Clinicians learn early on that their career will involve an endless grapple with an ever-growing, always incomplete universe of medical knowledge. As medical students, they learn to differentiate between uncertainty associated with lapses in their own knowledge and uncertainty related to gaps in medical science, which cannot be avoided (Fox 1957, 1980, 2000). But, recognizing uncertainty and addressing it in the clinic are two separate issues. Through clinical experience, physicians develop strategies for dealing with uncertainties that come to bear on diagnosis, treatment, and prognosis of medical problems and patients’ responses to them (Light 1979). At times, clinicians may recognize uncertainty but nevertheless hide it from patients in an effort to contain the threats that it poses to their professional medical authority (Fox 1957). These threats include, for instance, the use of uncertainty by parents of children being assessed for developmental disabilities to ground their resistance to clinicians’ diagnoses (Maynard 2003). Or, as in pediatric visits when the viral etiology of upper respiratory infections is likely rather than certain, parents can leverage the possibility of a bacterial infection to apply pressure for antibiotics and leave the visit with a prescription even when physicians orient to this as inappropriate (Stivers 2007).

### Uncertainty in Pediatric Neurology

Uncertainty is particularly at issue in pediatric neurology where nonneurological medical problems, such as syncope (i.e., fainting due to low blood pressure), tics, and even breath-holding spells, can sometimes mimic seizures (Fine and Wirrell 2020). For neurologists, this means that some patients may present with symptoms that look, to their families, like epilepsy but are actually unrelated to the condition. In fact, up to 20% of children who undergo vEEG monitoring are identified as having psychogenic seizures that would not benefit from antiepileptic drugs (Dhiman et al. 2014).

Although the incidence of pediatric epilepsy varies by syndrome and age, cumulative assessments suggest that it is the most common chronic neurological condition in pediatrics, affecting between .5% and 1% of children (Aaberg et al. 2017). While seizure control through medication is possible for most children (Camfield and Camfield 1996), approximately 20% will have seizures that are resistant to antiepileptic drugs (Wirrell 2013). Those who do achieve seizure control at one point in time might lose that control subsequently because their developing brains present a moving target for diagnosis and treatment. This study examines parents’ invocations of uncertainty in interactions with clinicians during inpatient visits for continuous vEEG testing. I focus on (1) where parents invoke uncertainty in their interactions with clinicians and (2) to what ends.

## Data And Methods

### Data

Data for this project came from a corpus of video-recorded medical visits for continuous vEEG monitoring. Data were collected in a pediatric neurology clinic in a teaching hospital located in Southern California in 2018. Visits generally lasted 24 to 48 hours and provided an uninterrupted log of the electrical activity in the patient’s brain and a concurrent video recording of the patient’s body. This corpus consists of three parts: (1) admission interviews, (2) neurology team meetings in the lab, and (3) the rounds phase where the team reports findings to the families. A total of 41 families participated in the study, yielding 35 complete “sets” of data (i.e., admission, lab, and rounds). Those patients with incomplete sets were excluded from this analysis. Encounters across each segment of the corpus ranged in length from 45 seconds to 55 minutes and collectively represent over 10 hours of audiovisual data.

The patients comprised 21 boys and 14 girls ranging in age from six months to 17 years. Although the age range was wide, the corpus skewed young, with half of all patients aged five years or younger. Nearly two-thirds of the families were White, and nearly one-third were Hispanic. Household income skewed wealthy, with nearly two-thirds making over $100,000 a year and almost one-third bringing home over $200,000 annually. Some cases included both parents, and some included one parent. All participants provided informed consent, and Institutional Review Board approval was secured in advance. Detailed transcriptions of the data were made using the Jeffersonian notation system (Hepburn and Bolden 2013).

### Methods

This study uses conversation analytic (CA) methods. CA has proven valuable in the pursuit of a systematic account of communication in medical care (Heritage and Maynard 2006). It has been used to delineate the overall organization of medical visits, the activities comprising each phase (Robinson 2012), and how these activities are collaboratively achieved by doctors and patients (Maynard and Heritage 2005). This research has made it possible to articulate interactional challenges with precision and to develop interactional interventions for medical visits as a means of, for example, improving vaccination rates (Robinson and Heritage 2014).

In line with CA methods, I approached the admissions and rounds phases of the data inductively, starting with cases where parents broached issues of uncertainty. I then proceeded iteratively to refine this collection as what counted as uncertainty for the purposes of this study became clearer. In what follows, I provide greater specification of this analysis.

### Analysis

Two aspects of the phenomenon of “invoking uncertainty” were relevant for this analysis. First, “invocation” was meant to convey how parents bring up uncertainty. Invocations ranged from highly direct means, such as “I can’t diagnose him. I don’t know what’s causing this,” which invoked uncertainty about the diagnosis, to statements that convey a desire to know, such as “I want to know if he needs to stay on it. . . . Is it working?” which invoked uncertainty about the effectiveness of the treatment. Second, “uncertainty” was meant to capture aspects of the diagnosis or treatment that were presently unknown or where knowledge of them was speculative. Thus, regardless of whether parents’ invocations were direct or indirect, if they oriented to one or more aspects of the patient’s diagnosis or treatment as uncertain, then they were included in the collection. This generated an initial collection of 52 cases from visits with 19 families in which a parent invoked uncertainty.

The present study focused on the primary interactional context wherein parents broached uncertain aspects of their child’s condition in these data—in accounting for their intrusions on medical authority. These cases constituted 85% (n = 44) of the collection. As I show, the use of uncertainty as an account for their conduct allowed parents to circumvent the attribution of fault to present parties and thereby minimize the potential for conflict arising from it.

## Results

### Invoking Uncertainty

The patient in Extract 1 is five-year-old Ollie. Last year, Ollie was lying in bed with his dad, Otis, when his legs suddenly began shaking. Otis grew concerned when Ollie said that he could not make his legs stop. Although the episode subsided, similar episodes happened occasionally over the next several months, prompting his parents to seek a neurological evaluation. After an initial vEEG, the neurologist suspected that Ollie’s episodes were indicative of benign rolandic epilepsy, a self-limiting condition that children usually grow out of within a few years. Although his seizures will likely go away as his brain develops, the neurologist prescribed an antiepileptic drug called Keppra, which Ollie has been taking twice daily for the past seven months.

Ollie has not had any obvious episodes since he started taking Keppra. However, his mom, Olivia, brought him back for vEEG testing because, among other things, she wants to know whether his episodes have stopped because the Keppra is working or because he has outgrown his seizures and no longer needs medication.

**Extract 1: P40-1a**

1 Nur: Did they say that they’re just (.) w atching him

2     overnight just to see what’s- what’s happening?

3 Mom: I requested this.

4 Nur: [Oh:.]

5 Mom: [ I ] asked for another EEG to see if he ne-

6   he’s been on this dr u g twice a day for seven

7 mo nths and I don’t- it’s got like s eventy

8   side effects or something,

9 Nur:  [ Oh::. ]

10 Mom: [And I wan]t to know if he needs to stay on it,

11   Is it g one? Is it working?

12 Nur: Ye:ah. Yeah.

13 Mom: Is he still having the s ame amount of seizure

14   activity at sl eep_? And we’re just giving him

15   this d rug. And we don’t even need to be, Or

16   he needs m ore, he needs no ne_

17   (0.4)

18 Mom: I mean (.) wouldn’t you want to know?

19 Nur: [ Yeah. Yeah. (.) Yeah. ]

20 Mom: [Rather than just g iving your] k id this m edicine

21   that has all these s i de effects that . . . .

22 Nur: Y eah.

23   (0.3)

24 Mom: That we may not even kno:w (0.3) [what ] it could=

25 Nur: [Yeah.]

26 Mom: =cause over yea:rs’ worth of t i:me_ And (0.4) I’m

27   just being thorough_

28 Nur: Yeah.

*Note*: Nur = Nurse. See Appendix for transcription notation meanings.

In Extract 1, Olivia invokes various uncertainties related to Ollie’s condition and the medication he has been taking for it. In asserting her desire to know whether Ollie needs to continue taking medication (line 10), Olivia treats the matter as uncertain. She expands her turn at line 11 with a pair of unanswerable questions that invoke uncertainty related to whether Ollie has outgrown his epilepsy (“Is it gone.”) and whether the drug is effective (“Is it working,”), thereby accounting for having asserted a desire to know whether he needs the medication. After the nurse acknowledges this (line 12), Olivia expands her turn with a second compilation of unanswerable questions delivered in rapid succession that, although stated differently than before, effectively invoke the same dimensions of uncertainty (lines 13–16). Subsequently, she invites the nurse to agree with her and thereby validate her desire to know—“I mean (.) wouldn’t you want to know,” (line 18). This overbuilt turn and the barrage of presently unanswerable questions that comprise it serve to invoke uncertainty about Ollie’s treatment.

Three features of this sequence are important for understanding what Olivia is doing when she invokes uncertainty: (1) The nurse’s expectation is conveyed implicitly in the form of a presupposition underlying a question she asks Olivia; (2) Olivia’s response rejects this presupposition, thereby breaching the nurse’s expectation; and (3) Olivia invokes uncertainty as an account for her breaching conduct. In what follows, each of these elements will be examined in turn.

### Presuppositions as a Window into Clinicians’ Expectations

The presuppositions underlying questions provide a first key form of evidence for what is viewed as expectable. Questions unavoidably advance propositions that themselves routinely impose presuppositions (Heritage 2010). For example, “What kind of contraception do you use?” presupposes not only that the recipient (1) uses contraception but also that (2) she is sexually active and (3) able to bear children but (4) does not want to get pregnant (Heritage 2010:47). Unless the recipient actively resists these presuppositions, thereby rejecting the validity of the question, her answer will ratify them (Ehrlich and Sidnell 2006; Heritage 2003). With this in mind, question design offers a window into clinicians’ expectations.

In Extract 1, the nurse is working to understand the physician’s rationale for the overnight testing. With her polar question at lines 1–2, the nurse puts forward a proposition for why Ollie’s neurologist might have ordered this, which is “just to see . . . what’s happening,”. Absent negative polarity items like *any* and *at all*, the grammatical construction of this question invites an affirming response—the nurse invites Olivia to confirm this as the plan (Heritage and Robinson 2011).

### Breaching Normative Expectations

Although declarative questions like the one issued by Ollie’s nurse invite unexpanded affirmative confirmations (Heritage 2010:49–50), Olivia builds an expanded response wherein she asserts an alternative explanation for the vEEG—that *she* requested it (line 3). This answer transforms the presupposition underlying the nurse’s question, rejecting the proposition she advances with it (Stivers 2022; Stivers and Hayashi 2010). Key for us is that the nurse’s proposition is built on the presupposition that the agency for the testing lies with the physician who has the epistemic authority to know and the deontic authority to determine whether and when testing is appropriate (Heritage and Raymond 2005, 2012; Stevanovic and Peräkylä 2012).

In this context, the nurse’s third position change-of-state token, “Oh:.” (line 4), appears to reveal her orientation to Olivia’s request for testing as a breach of normative expectations. Although this change-of-state token is not itself determinative of this, with it, the nurse orients to the presumption underlying her inquiry as mistaken and overtly registers Olivia’s response as corrective (Heritage 1984a). The nurse’s presupposition, Olivia’s response to it, and the nurse’s orientation to this response as corrective all embody their mutual orientation to normative expectations and departures from them.

### Accounting for Breached Expectations

In providing an account in this context (lines 5-8, 10–11, 13–16), Olivia shows that she recognizes her request for a vEEG as a departure from normative expectations and that doing so is a morally accountable matter (Garfinkel 1967). At lines 10–11, Olivia invokes uncertainty through asserting a desire to “know if he needs to stay on it, Is it gone. Is it working,”. She then uses this to build an extended account wherein she situates her conduct within a constellation of uncertainty, which furnishes the reasonable grounds for the breach (lines 13–16).

To situate her decision in a constellation of uncertainty, Olivia articulates a series of presently unanswerable questions through which she invokes a range of uncertainties, such as whether Ollie needs to continue taking Keppra (line 10) and whether he has outgrown his seizures (line 11) or the medication is inhibiting seizure activity as intended (line 11).

Even after the nurse produces acknowledgment tokens (line 12), Olivia expands her list with yet more unanswerable questions that invoke uncertainty related to whether Ollie is still having seizures despite the medication (lines 13–15) and whether they should be giving him more of the drug (line 16) or none at all (line 16).

Through invoking these uncertainties, Olivia invites the nurse to understand her conduct in light of the circumstances—within a constellation of uncertainty. This furnishes reasonable grounds for what might have appeared to be unreasonable conduct insofar as her decision to request testing is presented as an effort to protect her son, a mother’s obligation. In showing that there was justification for the breach, she invites the nurse to (re)interpret her conduct as reasonable.

Subsequently, at line 18, Olivia uses a negative interrogative “I mean (.) wouldn’t you want to know,”—which offers additional evidence of her orientation to her potentially problematic conduct as reasonable under the circumstances (Heritage 2002). In the context of complaints, “I mean” prefaced utterances have been shown to be deployed in the service of pursuing alignment and, as is the case here, serve to “skip-tie” back to the speaker’s prior utterance, thereby sequentially deleting the vacant response opportunity space at line 17 (Maynard 2012). Although Olivia is not using this in the context of complaining, her “I mean” prefaced negative interrogative (line 18) operates similarly to those discussed by Maynard (2012:219) in that it “essentially ignores a lack of responsiveness.” It also provides us with insight into what she is doing with her questions—building a case for the reasonableness of having requested the testing. Prior research on negative interrogatives suggests that they are the strongest form of yes-preferring question, and they are argumentative or “hostile” to the extent that they apply pressure for a response from the recipient that conflicts with their prior statements or actions (Heritage 2002). Here, Olivia’s negative interrogative is strongly positively polarized in a context where the nurse’s agreement would serve to undermine her prior orientation to Olivia’s conduct as unexpected and thus, potentially problematic. In this way, Olivia overtly takes a stance toward her rationale as reasonable and her conduct as righteous under the circumstances. In response, the nurse offers agreement (line 19), thereby affiliating with Olivia’s position and ratifying her conduct.

Extract 1 provided support for the claim that parents can invoke uncertainty as an interactional resource in environments where they have breached normative expectations associated with medical authority. Extract 2 offers further support for this claim. Here, we meet Linda and her three-year-old son Luke. Linda brought Luke in for testing because she noticed that he stares off and become unresponsive for short periods of time. This is particularly worrisome for Linda because her eldest son has autism spectrum disorder, and she fears that Luke might also be on the spectrum.

Extract 2 comes from the admission phase of the visit. Here, the resident is gathering Luke’s medical history and updating his electronic medical record. At the onset of this extract, the resident inquires about whether Luke’s vaccinations are up to date (line 1), and the breach in this case emerges with the revelation that they are not (line 4).

**Extract 2: P22-1a**

1 Res:  Is his Are his vaccinations up to date_?

2    (0.7)

3 Res:  His shots_=

4 Mom: =Mm:: (0.2) Probably not.

5   (0.2)

6 Res:  Oh, probably not?

7 Mom:  Probably not. Ye[ah.]

8 Res:          [Okay_]

9 Mom: Uh, because, uhm, my, uh, my my older son’s

10  uh, condition.

11 Res: Mmhm.

12 Mom: Uhm, either it’s ah true or no:t.=Uh, the

13   vaccine might cause it or not. And I

14   just wanna be more caution with him.=

15 Res: =So, is he on like a delayed [schedule?]

16 Mom:            [S:low]=I

17    yeah.

18 Res: [Got it.]

19 Mom: [ I wann]a do it slowly_ Yeah.

20   (3.0) ((Res faces computer and begins typing))

*Note*: Res = Resident.

In the context of the doctor–patient relationship, there is a preference for patients to accept physicians’ professional advice and validate their deontic authority. Patients infrequently resist physicians’ recommendations (Stivers et al. 2018; Thompson and McCabe 2018), and the majority of children receive most routine vaccinations by 24 months of age (Hill et al. 2021). Linda’s decision to delay Luke’s vaccinations at her own discretion constitutes a breach of this norm and a challenge to medical authority. It is within this breaching context that Linda invokes uncertainty about the safety of vaccinating her son on the standard vaccination schedule—mainly, that she fears it could cause autism (lines 12–14).

Extract 2 begins as the resident issues a positively polarized and thus yes-preferring, interrogative question (line 1). However, this gets no uptake from Linda (line 2). After a 0.7 second silence, the resident pursues a response, replacing “vaccinations” with “shots” and renewing the relevance of an answer from Linda (line 3). Her answer is delayed (with a filled pause “Mm” and a brief silence), mitigated (“Probably”), and dispreferred insofar as it inhibits progressivity (line 4). She accounts for this dispreferred answer first by reference to her older son’s “condition” (lines 9–10) and subsequently through invoking uncertainty about whether vaccines could cause Luke to develop autism (lines 12–14).

Thus, unlike Olivia in Extract 1, Linda does not take issue with the effectiveness of the medical intervention but with the ancillary problems that she fears it could cause. Although their procedures for invoking uncertainty vary, Olivia and Linda both use uncertainty in the service of accounting for their intrusions on medical authority.

Extracts 1 and 2 provide support for my claim that parents can invoke uncertainty as a resource in accounting for present conduct that somehow breaches normative expectations associated with medical authority. Extract 3 extends this claim, illustrating how parents can make strategic use of uncertainty in accounting for past intrusions on medical authority.

The patient is Tina, a three-year-old with suspected absence seizures. Last year, Tina was preparing to hit a piñata but suddenly stopped moving and became unresponsive with a blank stare. Roughly 30 seconds later, she suddenly resumed swinging as though nothing had happened. Staring spells like this began occurring every few days, which prompted Tina’s mom, Tori, to notify her pediatrician. Tina’s pediatrician suspected that her episodes were absence seizures and decided to refer Tina to a neurologist. During her first visit to the neurology clinic Tina had an outpatient EEG, the results of which were inconclusive. However, the clinician felt that the episodes Tori described were concerning enough to warrant starting Tina on Keppra.

In Extract 3, Tori reports that neither Tina’s father (lines 13–15) nor grandmother (lines 17–18) believe her staring spells are concerning. Nevertheless, Tori insisted on bringing her in for an overnight vEEG. In this context, Tori invokes uncertainty through expressing doubt about her own concerns—“I mean for a while I was like is it j(h)ust like £is it just me::,£” (lines 19–20). Through expressing self-doubt, she invokes uncertainty related to whether there is a diagnosable problem.

**Extract 3: P07-1a**

1 Res:  Oh, question I wanted to ask. I know Doctor

2    Sharron Hardy: had recommended the Keppra:,

3    (.) but it-you guys didn’t start that, right?

4 Mom:  Yeah: her-her dad is no:t (0.3) like

5 Res:  He doesn’t see all these little things

6   [so he’s not ]

7 Mom:   [Yeah, he think-] Yeah, he thinks that I’m over

8   <I mean I could s-I have video to prove it now.

9 Res:   [Yeah. ]

10 Mom:   [Right,] [    Uhm:   ]

11 Res:    [And w’ll what doe]s he say when you

12   show him that? [Has he noticed her doi-]

13 Mom:     [ He he he said ] that

14    (.) he saw it and he said (0.3) “I don’t see

15    anything. She’s fine.”

16 Res:  [I see. ]

17 Mom:  [I’m like] she’s like (.) n-And I showed it

18  to my mom too and they’re like (.) °sh-=↑I don’t

19   know:_ I mean for a while I was like, is it

20   j(h)ust, like, £is it [just me::,£ ]?

21 Res:             [It’s subtle.] So it’s hard.

22      [And you know h]er: like (.) and you know how=

23 Mom:   [   Okay:,   ]

24 Res:  =sh[e acts ] so you notice [it, ]

25 Mom:   [Ri:ght_]         [Yea-]

26 Res:  It’s subtle. But [ I think ] you’re doing all=

27 Mom:         [I mean, it’s]

28 Res:  =the right [things.]

29 Mom:       [ Yeah.]

*Note*: Res = Resident.

Like Olivia in Extract 1 and Linda in Extract 2, Tori invokes uncertainty in the context of a breach. However, in this case, the breach is a failure to adhere to the medication prescribed in the past. Recall that months prior to this visit, Tori began noticing Tina’s staring spells. This prompted Tori to arrange an outpatient visit with a neurologist who prescribed Keppra (lines 1–2). However, Tori evidently never filled the prescription (line 3) and thus failed to adhere to the clinician’s recommendation, thereby challenging her deontic authority.

When the resident broaches the issue of noncompliance (lines 1–3), Tori responds with a confirmation followed by the onset of an account, which she displays some difficulty formulating (line 4). Although she stops short of explicitly shifting blame for the breach onto her husband Tim (e.g., “her dad is no:t” [convinced]), she nevertheless implicates parental disagreement as a contributing factor. The resident registers this (lines 5–6), offering a candidate account that centers on Tim’s failure to align with Tori. Within this context, Tori begins to invoke uncertainty through expressing self-doubt (lines 18–20). When Tori says “I mean for a while I was like is it j(h)ust like £is it just me::,£” (lines 19–20), she uses the possibility that Tina’s behavior is normal to account for her failure to follow medical advice. If there is a chance that Tina’s episodes are benign, then holding off on starting medication is reasonable, and her apparent intrusion on medical authority is warranted.

So far, I have shown that parents can invoke uncertainty as a resource in contexts where they have breached normative expectations associated with their role in the child’s care. One question at this juncture is: What can help explain the use of uncertainty in accounting for conduct that threatens clinicians’ professional medical authority? What advantages might uncertainty-based accounts have over alternatives? In the following section, I argue that uncertainty accounts offer two key advantages: (1) They have a “no-fault” quality, and (2) they minimize conflict. Together, these advantages help promote progressivity of the visit and mitigate damage to the physician–family partnership. Moreover, as we will see, parents can use uncertainty accounts as a vehicle for displays of identity (e.g., *doing being* a good mom, Sacks 1984; see also, Heritage and Sefi 1992; Voysey Paun 1975). This works, in part, by making parents’ conduct intelligible as reasonable *under the circumstances*.

### Advantages of Uncertainty Accounts

There are unique advantages to parents’ invocations of uncertainty in challenges to medical authority. To better illustrate these advantages, let us first examine a case wherein the patient’s mother does not invoke uncertainty as an account for her intrusion on medical authority. For this, consider Extract 4: The patient, 16-year-old Jay, has briefly lost consciousness on four occasions in the past three years. Her mom, Jessica, insisted on bringing Jay in for a vEEG despite three separate clinician diagnoses of Jay’s episodes as syncope (i.e., fainting related to low blood pressure). Jessica’s insistence on pursuing testing despite receiving multiple nonseizure diagnoses represents a challenge to epistemic and deontic dimensions of medical authority.

Extract 4 is taken from the rounds phase of the visit, after the resident reports that Jay’s vEEG results were inconclusive: Jay did not have an episode during the observation period, but her EEG did reveal abnormal electrical activity in her brain. Although this abnormal activity was insufficient for diagnosis, it could indicate a higher than average seizure potential. Consequently, the team requests that Jay stay for a second night so they can try to capture an event, which will allow them to determine a diagnosis and treatment. Orienting to this as some degree of vindication, Jessica recasts her breaching conduct as necessary due to the faulty judgment of one present and two nonpresent clinicians. Rather than invoke uncertainty as an account for her encroachment on medical authority as in Extracts 1 through 3, Jessica blames the neurology fellow standing in front of her (lines 5–8). Although direct conflict such as this is exceedingly rare in these data, it is nevertheless illustrative of how conflict can arise in complex medical contexts.

**Extract 4: P38-1r**

1 Mom:    Oh, we saw you: (points to fellow)

2 Res:    Yea:h.

3 Mom:   Right. We saw you.

4 Fel:     [(In May)]

5 Mom:    [And you:] (.) thought it was nothing.

6       (0.4)

7 Mom:   Like you just sent us on our way and said “Don’t

8      worry about it.” And then when we went back this

9      time (.) we went to our pediatrician and she was

10     like (.) “Eh, don’t worry about it.”

11       (.)

12 Mom:  And then we went to see Doctor Crew and I had tuh

13       (.) demand (.) I had to sort of say ↓no: >you know<

14      So, (.) literally none of this would be happening

15     were it not for me:_

16       (.)

17 Mom:   Like no:body at any point (.) said (.) “Come to the

18     hospital and be worried about this.”

19 Att:      Well, en #I-# I think (0.7) they could-we could still

20     be (.) dealing with two different things, One being

21      the orthostatic, (0.3) uhm, symptoms and the syncope?=

22 Mom:  =Right.

23 Att:  So, the fainti:ng. .h And then she has this abnormal

24        finding on her EEG.

25 Mom:  Right. I’m [just saying thee] EEG would never=

26 Att:    [ That might be ]

27 Mom:  =have happened [ had ] I not said (.) “I’d like=

28 Att:    [Right.]

29 Mom:   =tuh have an EEG.”

30       (0.3)

31 Mom:  I mean ↓nobody: no doctor we’ve ever seen (0.2)

32     expressed (0.7) any (0.2) sort of interest.

33       (.)

34 Mom:  In pursuing any:thing about th[is.]

35 Att:    [<Be]cause clinically it

36     doesn’t sound like seizures. <So (.) if sh-(0.4) so if

37      we hadn’t seen that on the- <So if we hadn’t see:n the

38     EEG without just (0.2) without doing the kind of light

39       (.) thing that we [did] with her, (0.3) then we would have=

40 Mom:                [Mhm]

41 Att:  =said (1.0) go home today.

42 Mom:  Mkay_

*Note*: Att = Attending; Fel = Fellow; Res = Resident.

Jessica’s attribution of blame creates a conflict-prone interactional environment that inhibits progressivity of the visit: After confirming that she and Jay saw this neurology fellow previously (lines 1–3), Jessica asserts “And you: (.) thought it was nothing.” (line 5). Two key elements of this formulation prime the interaction for conflict: First, Jessica’s use of contrastive stress on “you:” invokes the opposite (“me”), which sets up an adversarial stance in that it casts the fellow’s view as one that she does not share. That is, YOU thought it was nothing, but I did not agree.

Second, Jessica’s use of “thought” attributes fault to the fellow because her past-tense formulation implies that the fellow’s belief then is not consistent with the medical team’s present assessment (i.e., the fellow was wrong). When her turn gets no uptake at line 6, Jessica expands with a characterization of the fellow’s conduct as dismissive—“Like you just sent us on our way and said don’t worry about it.” (lines 7–8). Insofar as her previously unmet concerns have now been validated, the fellow’s no-problem dismissal of Jessica’s concerns is presented as an accountable matter. Through foregrounding these elements, Jessica takes an adversarial stance vis-à-vis the fellow and thereby establishes the basis for confrontation.

At this point, Jessica expands the scope of her grievance to include Jay’s pediatrician (line 9) and a nonpresent member of the neurology team (line 12). In detailing her prior encounter with Dr. Crew, Jessica asserts that she “had tuh (.) demand (.)” further consideration of Jay’s episodes (lines 12–13). Subsequently, she presents herself as someone who had to overcome multiple clinicians’ professional advice as one after another attempted to dismiss her concerns (lines 14–15). Finally, when no uptake appears to be forthcoming at line 16, Jessica uses multiple extreme-case formulations to legitimize her grievance (Pomerantz 1986). She asserts that “no:body” (line 17) at “any” (line 17) point along Jay’s diagnostic odyssey aligned with her as an ally. In positioning herself in opposition to the fellow in the first instance and to all of Jay’s prior clinicians subsequently, Jessica has created a conflict-prone context where opportunities for disagreement far outnumber opportunities for agreement.

The attending attempts to avoid disagreement through invoking the possibility that Jay’s past episodes were distinct from her current episodes (lines 19–21, 23–24). However, Jessica resists the attending’s move, responding with a revival of her initial adversarial characterization (lines 25, 27, 29) followed by a variation of her prior extreme-case formulations (lines 31–32, 34). At this point, the attending launches an account—“<Because clinically it doesn’t sound like seizures.” (lines 35–36)—with a rapid onset indicated by the “<” that makes it sound rushed and thus, in this context, defensive. Its subsequent unpacking is marred by self-repair as the attending displays some difficulty in exiting the sequence (lines 36–39).

This case offers two key insights: (1) Through her attribution of fault to the clinician as an account for her breaching conduct, Jessica primes the context for conflict, and (2) the absence of agreement inhibits progressivity in the visit. To understand how these insights are related, consider Whalen, Zimmerman, and Whalen’s (1988) notion of *activity contamination*: Once participants enter contexts of conflict, the activity that was underway previously becomes “contaminated” by it. For instance, parents are more likely to resist nonantibiotic treatment recommendations when pediatricians explicitly rule out antibiotics as an option (Mangione-Smith et al. 2006). Heritage (2011:342) argues that this is an artifact of activity contamination, which pediatricians occasion “by casting parents as having wanted an antibiotic prescription all along and, in the very same moment, rejecting that treatment preference as inappropriate.” In these interactional contexts, conflict becomes the frame of reference for conduct—assertions of fact are not heard as factual information but as moves within conflict. This is evidently the case in Extract 4 when the attending broaches the possibility that there could be two distinct medical problems (lines 19–21, 23–24). Jessica orients to this not as a piece of factual information but as a counter move within an ongoing dispute, thus thwarting progressivity of the visit (lines 25, 27, 29, 31–32, 34).

By contrast, uncertainty accounts, like inability accounts, offer a “no-fault quality” (Heritage 1984b: 271–72; 1988). Parents can use uncertainty to frame their breaching conduct as the unavoidable consequence of uncertain circumstances. This makes for an interactional environment that minimizes flash points, such as blame sequences, that can serve as catalysts for conflict. In Extract 2, for instance, Linda could have grounded her reluctance to vaccinate in an accusation against physicians for risking harm to her son. However, doing so would have made conflict more likely and agreement more difficult. Instead, Linda invokes uncertainty as an account for her breaching conduct—“Uhm either it’s ah true or no:t. = Uh the vaccine might cause it or not. And I just wanna be more caution with him.” (lines 12–14)—circumventing the attribution of fault and minimizing the potential for conflict.

At times, the no-fault character of uncertainty accounts can even allow for greater alignment and affiliation than would be possible with other accounts. In these cases, parents and clinicians can unite against uncertainty rather than act as adversaries. To better illustrate this distinction, let us return to Tori, whom we first met in Extract 3. Extract 5 comes from the final minutes of the admission phase of the visit.

**Extract 5:P07-3a**

58 Res:  And, uhm, [then, we’ll make some more decisio-]

59 Mom:           [ And it’s good. Right_ ]

60     >I mean< (.)

61 Res:  .tch This isn’t hurting her.

62 Mom:  >Y-no< I gu[ess ]

63 Res:           [Yeah.]

64 Mom:  I guess I’m asking because (.) I think

65      from like (.) hearing (0.4) from her da:d

66      (.) and my mom like (.) Well, my mom

67        actually said “Yeah. Take her.” But her dad was

68       like (.) you know you’ll upset her. I feel like

69       I’m just doubting the fact that I brought her

70        but [it’s good that I brought her.]

71 Res:   [ <I think it’s a great thing ] that you

72     brought [her. ]

73 Mom:          [Okay.]

*Note*: Res = Resident.

Here, Tori invites the resident to agree with a positive characterization of her breaching conduct—“And it’s good. Right_” (line 59). While the resident’s response does not overtly disagree (line 61), her transformative answer (“This isn’t hurting her.”) elides the question’s topic and action constraints (Stivers and Hayashi 2010). Rather than agree that Tori’s pursuit of testing is good, despite having already been assessed and prescribed previously, the resident adopts a no-harm position. She neither ratifies Tori’s breaching conduct nor condemns it. In this context, Tori reinvokes uncertainty through expressing self-doubt (line 69) and recasts her breaching conduct as positive (line 70). This time, however, the resident produces an upgraded assessment of Tori’s conduct—“<I think it’s a great thing that you brought her.”—from “good” to “great” (lines 71–72).

In using uncertainty as an account, Tori specifically avoids attributing blame to present parties just as she avoids assuming it herself. Ultimately, uncertainty accounts threaten neither the “face” of the parties involved nor their social relationships (Goffman 1982). Thus, parents can use uncertainty to challenge medical authority indirectly, under the guise of caution, thereby exploiting their legitimate caretaking role. This no-fault framing minimizes conflict by avoiding moral accountability for parental intrusions on medical authority and allows visits to proceed on the basis of a presumed commonality of concern for the child’s best interests. Unlike Jessica’s fault-driven account, Tori’s uncertainty account does not inhibit progressivity of the interaction. Rather, it minimizes disagreement and thereby provides for advancement of the visit.

Aside from promoting progressivity, uncertainty invocations appear to be one of the mechanisms by which participants can make particular identities relevant and consequential in this context (Raymond and Heritage 2006). During these encounters, parents can be observed grappling with multiple and sometimes conflicting vectors of normative constraint on their conduct. Whereas on the one hand parents assume the role of mom or dad, on the other hand, they assume the role of patient-by-proxy. They are tasked with following medical advice as patients-by-proxy and with ensuring due diligence in protecting their children, a parent’s obligation. The use of uncertainty as an account allows parents to manage these conflicting vectors of normative constraint by circumventing the attribution of blame to present parties and thereby creating a face-preserving interactional context that is resistant to conflict but hospitable to affiliation and alignment. Moreover, parents can use uncertainty accounts as an opportunity to *do* being a good parent.

The identity implications of these no-fault accounts can be observed if we return to Olivia from Extract 1. The following fragment comes from just after what was shown in Extract 1. Here, Olivia invokes uncertainty through asserting a desire to know more about the present state of Ollie’s condition (lines 2–3) and casts her breaching conduct as motivated by her desire to be a good mother—“↑just want to be a good momma_” (line 16).

**Extract 6: P40-3a**

1 Nur:  [Yeah.]

2 Mom:  [ So, ] (.) I just want answers_ I wanna know

3      what’s goin’ on with his little brain_ He’s thriving,

4      He’s (0.3) s:uper athletic, He’s eats great food,

5      kale salads ‘nd (0.3)

6 Tec:   Da:ng kale,

7 Mom:   We do [sugar but not a lot of sug]ar,=

8 Nur:       [ That’s all good science_ ]

9 Tec:   =Yeah.

10 Nur:   Yeah.

11 Mom:  Yea::h, I mean he’s- (.) his sister will be here soon.

12     She’s a toddler. She’ s-she’s an animal.

13       (0.3)

14 Mom:   But, uhm: (0.5) yeah, I mean he’s (.) he’s ra:d and I

15     just want to catch: anything_ If there’s more or if

16     there’s (0.2) ↑just want to be a good momma_

17      [(H)eh]

18 Nur:   [Aww::]

19 Tec:   Yeah.

20 Nur:   You’re doing gr(h)eat_

*Note*: Nur = Nurse; Tec = Technician.

In this case, Olivia uses uncertainty as a means of displaying her identity as a “good” parent (cf. Heritage and Sefi 1992; Voysey Paun 1975). She invites the nurse to understand her conduct in terms of this identity—as motivated by the desire to “be a good momma_” (line 16). Here, as was the case with Tori in Extract 3, Olivia’s account appeals to parental rights and obligations. Her no-fault framing minimizes disagreement and ultimately gets affiliative uptake from the nurse, who produces an empathic interjection—“Aww::”—at line 18, followed by an explicit ratification of Olivia’s maternal identity and the conduct she has attributed to it (line 20).

Although Olivia’s case presents an explicit display of the identity work brought off with invocations of uncertainty, implicit displays can be observed in other cases as well. As we saw in Extract 3, the resident orients to Tori’s uncertainty account in terms of its identity implications when she reassures Tori about her decision to pursue care despite her husband’s view of Tina’s conduct as normal—“It’s subtle. So it’s hard. And you know her: like (.) and you know how she acts so you notice it,” (lines 21–22, 24). In this way, identity adds meaning to the breach.

## Discussion

This article began with a rough sketch of “invoking uncertainty” as an interactional phenomenon. Analysis of the position and design of instances in the collection revealed that parents can use uncertainty invocations in accounting for conduct that breaches normative expectations associated with medical authority. After establishing where and to what ends parents invoke uncertainty, consideration was given to why parents might use uncertainty rather than something else in accounting for their conduct. I found that uncertainty accounts offer a no-fault quality that can help minimize conflict between parent and doctor unlike fault-driven alternatives such as blaming the clinician. Invoking uncertainty in the context of a breach allows parents to circumvent blame by treating the breach as a product of uncertain circumstances. These findings extend prior research on parents’ use of uncertainty to ground their resistance to diagnoses (Maynard 2003) and treatment recommendations (Stivers 2007), thereby leveraging it to influence visit outcomes. Moreover, it shows us how participants can use uncertainty as a means of displaying particular identities in specific contexts.

Parents can use uncertainty to challenge medical authority indirectly, under the guise of caution. Yet in using uncertainty to account for their encroachment on medical authority, parents ratify its legitimacy. While these breaches suggest that medical authority can indeed come under siege (Stivers and Timmermans 2020), parents’ treatment of them as accountable embodies respect for clinicians’ professional authority. Thus, while being a “good” patient was once thought to entail being a passive recipient of care (Freidson 1970; Parsons 1951), being a “good” patient today entails being engaged in one’s care (Timmermans 2020) but doing so in ways that still respect the physician’s role. As I have shown, the use of uncertainty as an account for conduct that encroaches on medical authority allows parents to enact their own role as “good” parents while also respecting the authority of medical professionals.

Much of the prior work on uncertainty in medicine emphasized the threats that uncertainty poses to the doctor–patient relationship (e.g., Fox 1957, 1980, 2000). Yet, as we have seen, and consistent with prior conversation analytic work on uncertainty in medical contexts (e.g., Gill 1998; Pilnick and Zayts 2014), uncertainty is not always a liability. In the cases presented here, parents use uncertainty as a resource in accounting for conduct that encroaches on medical authority. In this context, uncertainty is leveraged as an asset in the preservation of physician–family partnerships.

### Limitations

There are several limitations to the present study that warrant consideration. The analysis presented here is based on a limited collection of instances, which precludes appreciation of the full range of practices that uncertainty can be invoked to achieve. Moreover, of the 52 cases that comprise the initial collection, only 44 were included for analysis. The remaining 8 cases involve the invocation of uncertainty as a means of accomplishing other types of work, such as asking questions indirectly. There are undoubtably many ways that parents can invoke uncertainty and many ways that they can use it in interaction. The present study focuses exclusively on the primary usage observed in these data.

The data for this study were collected in the United States, where health care is primarily a fee-for-service enterprise. Claims regarding participants’ orientations to relative rights and expectations might not hold for countries that have adopted a single-payer health care system. Moreover, the families who agreed to participate in the present study were largely White and wealthy. Further work with greater diversity among participants with respect to race, socioeconomic status, and region of residence would be necessary to test the robustness of these findings.

## Appendix

### Transcription Notation

Jeffersonian Transcription Conventions

Adapted from Hepburn and Bolden (2013)

**Table:**

[ ]	Square brackets show beginning and ending of overlap in speakers’ utterances.
=	Equal signs indicate that utterances are latched.
(0.5)	Numbers in parentheses are silences timed to tenths of a second.
(.)	Period in parentheses is a very brief silence (less than two-tenths of a second).
((horn honks))	Transcriber’s comments are enclosed in double parentheses.
( )	Empty parentheses denote indecipherable utterance.
(text)	Text in parentheses is transcriber’s “best guess” as to a speaker’s utterance.
word_	Underscore following an utterance indicates level intonation.
.	Periods indicate strong falling intonation.
,	Commas indicate slightly rising intonation.
?	Question marks indicate strong rising intonation.
:	Colons indicate that a sound is stretched. The more colons, the longer the sound.
.hh	“h”s with preceding period indicate audible in-breath; the more “h”s, the longer the in-breath.
WORD	Upper case indicates especially loud sounds relative to the surrounding talk.
every	Underlines indicate parts of words that are stressed.
n-	Dash indicates a cutoff of a sound.
(h)	Parenthesized “h” indicates plosiveness, often associated with laughter, crying, breathlessness, etc.
>word<	Talk inside carets are spoken more quickly than surrounding talk.
°word°	Talk inside degree signs are spoken more quietly than surrounding talk.
#word#	Talk inside number signs is low-pitch/“creaky.”
↑	An upward-pointing arrow indicates a sharp upward change in pitch.
